# Characteristics of HPC(A) product obtained from a donor with SARS-CoV-2 infection and outcome of autologous transplant

**DOI:** 10.1186/s13287-024-03872-4

**Published:** 2024-08-13

**Authors:** Pranav S. Renavikar, Phyllis I. Warkentin, Shelly M. Williams, Krishna Gundabolu, Charles Branson, Scott A. Koepsell

**Affiliations:** 1 Department of Pathology and Microbiology, University of Nebraska Medical Center, Omaha, NE 68198 USA; 2 Department of Internal Medicine, Division of Hematology and Oncology, University of Nebraska Medical Center, Omaha, NE 68198 USA; 3 Department of Pathology and Microbiology, University of Nebraska Medical Center, Nebraska Medical Center, Omaha, NE 983135, 68198-3135 USA

**Keywords:** Hematopoietic progenitor cell, Covid-19, SARS-CoV-2, Apheresis

## Abstract

Collection of hematopoietic progenitor cell products [HPC(A)] is deferred if the donor is symptomatic and tests positive for Covid-19. However, donor questionnaires are subjective and may miss minimally symptomatic donors. Alternatively, myalgia associated with Covid-19 infection can be falsely dismissed as an adverse effect of granulocyte stimulating factor (Filgrastim) administered prior to product collection. The likelihood of donors with an underlying acute but minimally symptomatic infection undergoing successful product collection is significant. In these circumstances, it is less known whether Covid-19 infection results in product viremia or alters the clinical outcome of transplant. We aimed to evaluate the above question by studying a donor whose product was collected during acute Covid-19 infection. Aliquots of the product tested negative for SARS-CoV-2 RNA by reverse-transcriptase polymerase chain reaction assay (RT-PCR). Importantly, the donor received an autologous stem cell transplant using the product collected at the time of infection, and their case will be described in this report. We describe one of the very few reports of successful transplant of HPC(A) product collected during acute Covid-19 infection.

## Case presentation

An older male with relapsed grade 2 follicular lymphoma presented to oncology for consolidative high-dose therapy (BEAM) followed by autologous stem cell rescue (HDT-ASCR). The plan was to collect stem cells in the apheresis clinic, followed by immediate inpatient admission to begin consolidative therapy. The patient received daily injections of filgrastim (10 mcg/kg) without complication for five days before donation. The peripheral blood CD34 cell count prior to collection was 56/ul. The patient was completely asymptomatic, the vital signs were unremarkable, and he had no respiratory symptoms. The patient had completed two doses of the Pfizer-BioNTech Covid-19 vaccine. The hematopoietic stem cell collection proceeded using Cobe Optia. The total volume processed was fifteen liters, while the volume collected was 197 ml. The product was handled according to universal precautions for biologics, cultured for aerobic and anerobic bacteria and fungi, and cryopreserved according to standard operating procedures. Total CD34-positive cells collected after the procedure were 7.429 × 10^6^/kg. Patient tolerated the procedure well with stable vitals.

The nasopharyngeal swab for Covid-19 testing was obtained post-procedure as a routine pre-admission protocol. The results of the test identified SARS-CoV-2 RNA (cycle threshold value 22/22), thus delaying his admission for autologous transplant. He was administered sotrovimab (monoclonal antibody against SARS-CoV-2) two days post diagnosis. Repeat testing at two-week intervals showed a negative value on the third test. He subsequently recovered at home.

With the donor’s permission, the aliquot of the HPC(A) product collected at the time of acute Covid-19 infection was thawed, and total nucleic acid was extracted (five days post collection). RT-PCR was performed using primers for the SARS-CoV-2 E gene and the internal control RNase P gene (Applied Biosystems QuantStudioDx RT-PCR). Appropriate positive (EXACT Diagnostics EDX SARS-CoV-2 RNA standard) and negative controls were assayed. The RNase gene was amplified with a cycle threshold of 18.7. No Covid-19 RNA was detected after 40 cycles.

**Fig. 1:**
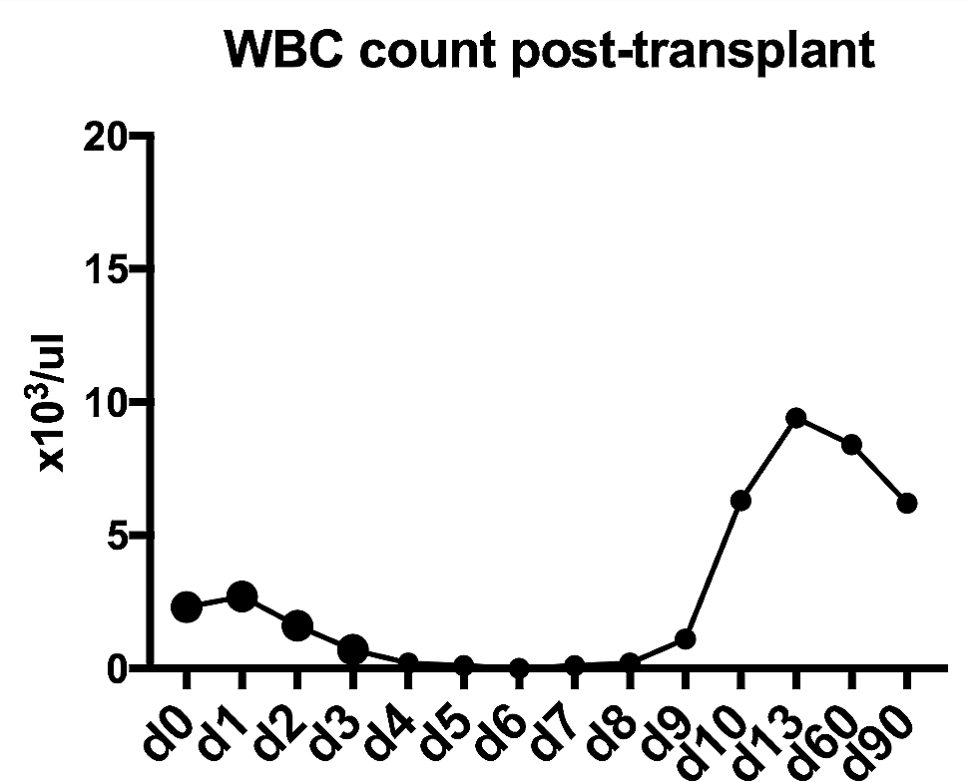
Recipient engraftment trend post-autologous transplant

After a delay of forty-five days since the first diagnosis of Covid-19, the patient was readmitted for HDT-ASCR. The BEAM protocol was administered without complication from day − 6 to day − 1, followed by autologous stem cell transplant on day 0. Patient received 240 ml of autologous stem cells (12.53 × 10^9^ nucleated cells and 43.38 × 10^6^ CD34-positive cells with 85.18% viability). He was started on prophylactic anti-viral and anti-fungal agents. The patient had chemotherapy-induced nausea, vomiting, anorexia and neutropenia on day + 4. G-CSF was administered on day + 7, and signs of count recovery were seen on day + 10. He engrafted well and without complication. Antibiotics were discontinued at discharge on day + 11. The patient’s white blood cell (WBC) trends are shown in Fig. 1.

## Discussion

In the post-pandemic setting, stem cell product donors may be entirely asymptomatic, not accurately represent their symptoms, or have filgrastim-associated myalgia overlap with Covid-19 symptoms. Sensitive PCR tests for Covid-19 can be persistently positive for weeks after infection[1]. This may indicate long-survival of viral nucleic acid in the nasopharynx. The asymptomatic Covid-19-positive patient is not likely to be a major source of new infections[2]. Covid-19 viremia has been reported in rare patients with severe, advanced infection but not in asymptomatic blood donors [3]; while weak positive RNA was identified in few pre-symptomatic donors[4]. Practically, the risk of transfusion transmission of SARS-CoV-2 is considered negligible[5].

For our donor, product collection had already occurred by the time of the positive test results of the Covid-19 nasopharyngeal swab. Most centers reportedly have not used HPC(A) products collected from such donors. A prior multi-institutional survey showed three allogeneic transplants with no viral transmission reported, and two autologous transplants[6]. Thus, data is lacking regarding testing HPC(A) products from Covid-19-positive donors prior to transplant, as well as outcome of transplant. We addressed whether Covid-19 RNA can exist or be transmitted in an HPC(A) product. The product collected from the positive donor did not contain any Covid-19 RNA above the limit of detection of the assay. The product was tested within five days of collection, which counteracts the possibility of decreased/loss of viability of the virus during PCR testing. Further, there exists a possibility of the donor being immune to the virus by the time of autologous transplant. However, given the aggressive myeloablation, immunosuppression, and risk for reinfection, the uncomplicated post-transplant clinical course was interesting.

Limitations to our report include that no assay is approved to test HPC(A) products. This testing was done using an assay that included an internal RNA integrity and RT-PCR control to ensure that the lack of detection of Covid-19 RNA is not due to loss of RNA quality or inhibitors of the PCR process. We did not have peripheral blood samples from the donor at the time of the HPC(A) donation, which would have helped to corroborate these results.

## Conclusion

HPC(A) product from an asymptomatic Covid-19 infected donor did not contain detectable virus, and the donor underwent successful autologous transplant. Our case underscores the potential for donation in minimally symptomatic cases and adds evidence for testing and use of the product if the risk versus benefit of a transplant warrants.

## Data Availability

Not applicable.

## Abbreviations

SARS–CoV–2: Severe acute respiratory syndrome coronavirus 2
HPC(A): Hematopoietic progenitor cell products collected by apheresis
Covid–19: Coronavirus disease 2019
RT–PCR: Reverse transcriptase polymerase chain reaction
RNA: Ribonucleic acid
BEAM: Carmustine, etoposide, cytarabine, melphalan
HDT–ASCR: High dose therapy and autologous stem cell rescue
G–CSF: Granulocyte colony stimulating factor
